# Supplementary material for: Anti-TNF Biologicals Enhance the Anti-Inflammatory Properties of IgG N-Glycome in Crohn’s Disease
Source: Biomolecules. 2023 Jun 7;13(6):954. doi: 10.3390/biom13060954 (PMC10295852; doi:10.3390/biom13060954)
Supplement: Supplementary file 1 [file biomolecules-13-00954-s001.zip › biomolecules-2419920-supplementary.pdf]

Supplementary Data

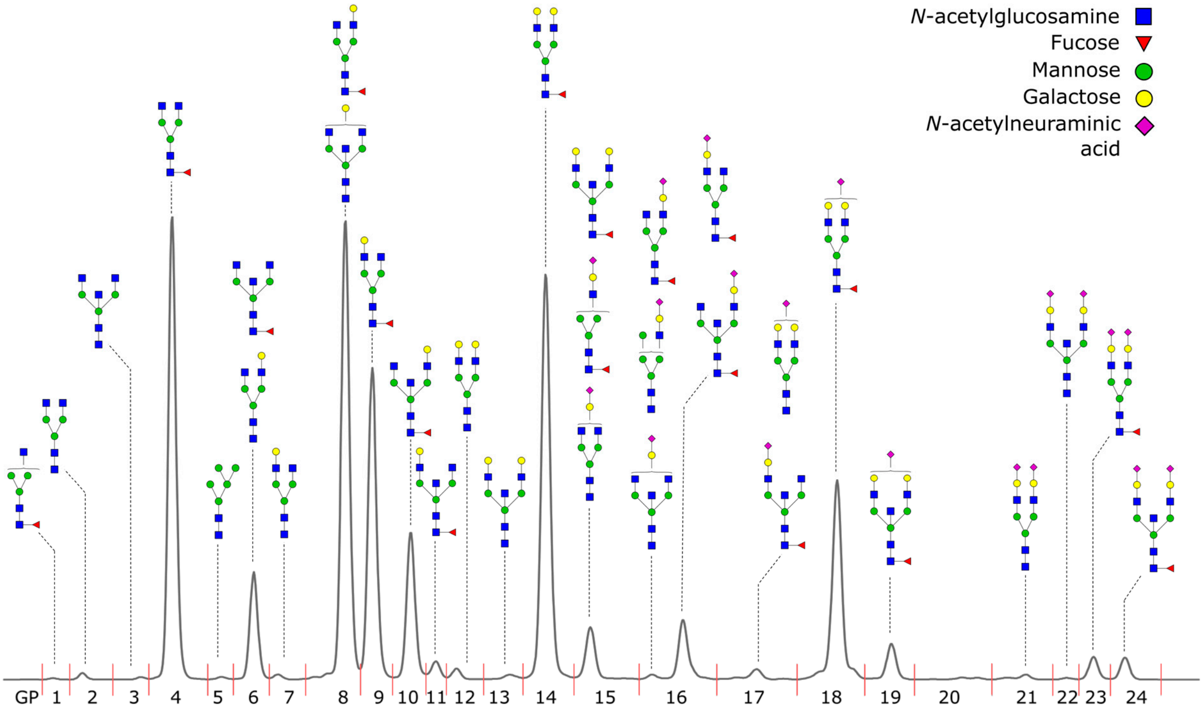

Figure S1. Representative HILIC-UHPLC-FLD N-glycoprofile of total serum IgG, separated into 24 glycan peaks (GP1 - GP24) and their corresponding compositions.

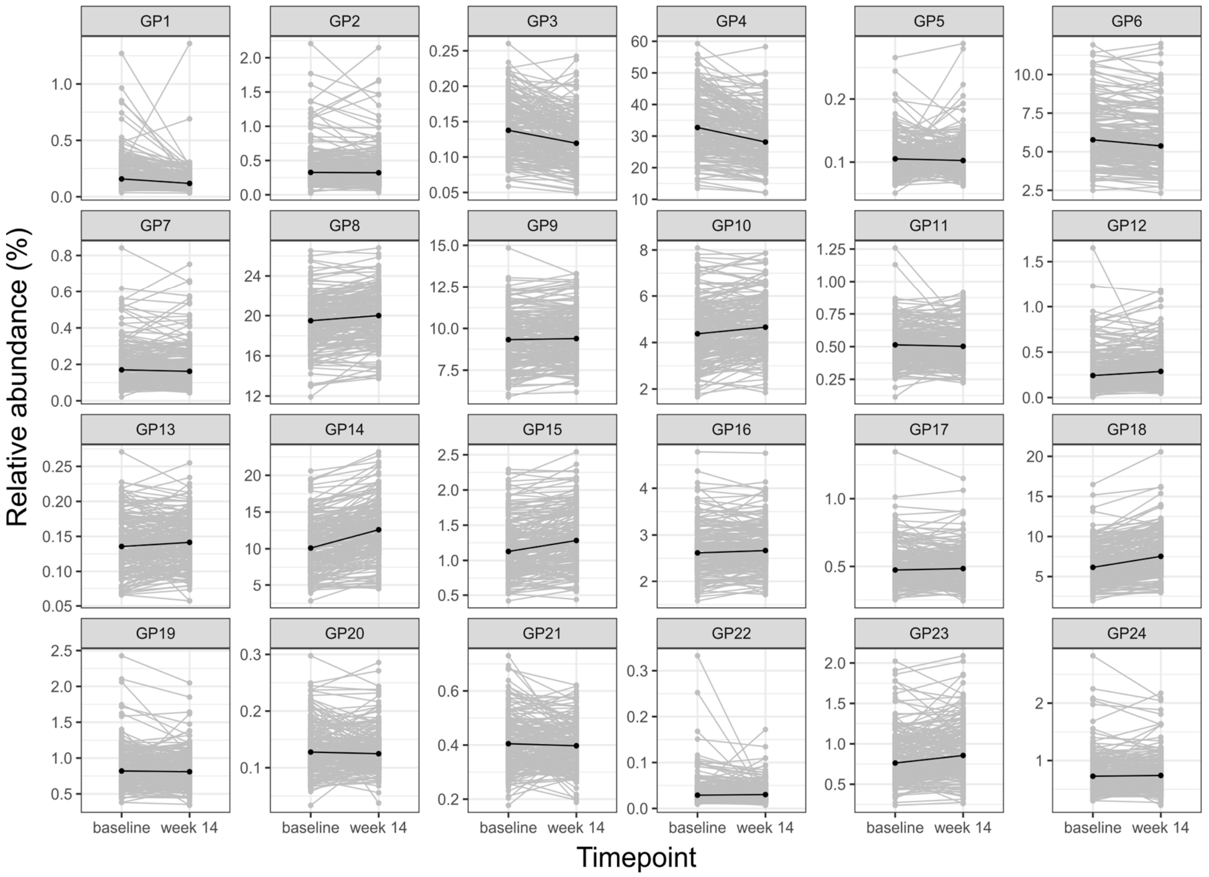

Figure S2. Changes in the relative abundance of directly measured IgG N-glycans in CD patients during the anti-TNF treatment (both IFX and ADA included). Each CD patient is represented with a single line. Median glycan values for each time point are bolded.

X-axis – timepoint (baseline, week 14), Y-axis – relative amount of individual glycan peaks (GP1-GP24, presented in Figure S1) expressed as a percentage (%) of total IgG N-glycome.

Table S1. Pairwise associations of derived IgG N-glycan traits at baseline with therapy outcome. Effect: the difference of a derived glycan trait (expressed in standard deviation units) between groups of CD patients regarding different therapy outcomes. *p*-values were adjusted for multiple testing and considered significant if <0.05 (bold).

Derived trait	Comparison	Effect	Standard error	*p*-value	Adjusted *p*-value
G0 total	PNR vs. Grey zone	-0,08	0,08	2,83E-01	4,07E-01
G0 total	PNR vs. Response	**0,28**	0,08	7,15E-04	**2,86E-03**
G0 total	Grey zone vs. Response	**0,38**	0,09	1,08E-05	**7,81E-05**
G0 total	PNR vs. Remission	-0,14	0,07	3,72E-02	7,05E-02
G0 total	Grey zone vs. Remission	-0,04	0,07	6,06E-01	6,94E-01
G0 total	Response vs. Remission	**-0,47**	0,08	1,02E-09	**1,99E-08**
G1 total	PNR vs. Grey zone	0,12	0,08	1,55E-01	2,43E-01
G1 total	PNR vs. Response	**-0,30**	0,09	5,78E-04	**2,60E-03**
G1 total	Grey zone vs. Response	**-0,40**	0,09	1,39E-05	**8,36E-05**
G1 total	PNR vs. Remission	**0,19**	0,07	9,54E-03	**2,64E-02**
G1 total	Grey zone vs. Remission	0,07	0,08	3,77E-01	4,85E-01
G1 total	Response vs. Remission	**0,50**	0,08	1,66E-09	**1,99E-08**
G2 total	PNR vs. Grey zone	0,11	0,08	1,36E-01	2,23E-01
G2 total	PNR vs. Response	**-0,26**	0,08	1,70E-03	**6,11E-03**
G2 total	Grey zone vs. Response	**-0,38**	0,09	8,06E-06	**7,26E-05**
G2 total	PNR vs. Remission	**0,17**	0,07	1,33E-02	**3,28E-02**
G2 total	Grey zone vs. Remission	0,03	0,07	6,41E-01	6,94E-01
G2 total	Response vs. Remission	**0,47**	0,08	1,53E-09	**1,99E-08**
S total	PNR vs. Grey zone	0,04	0,08	6,37E-01	6,94E-01
S total	PNR vs. Response	**-0,20**	0,09	2,06E-02	**4,64E-02**
S total	Grey zone vs. Response	**-0,26**	0,09	3,02E-03	**9,90E-03**
S total	PNR vs. Remission	0,09	0,07	2,34E-01	3,51E-01
S total	Grey zone vs. Remission	0,04	0,08	5,90E-01	6,94E-01
S total	Response vs. Remission	**0,33**	0,08	4,59E-05	**2,36E-04**
B total	PNR vs. Grey zone	0,03	0,07	6,55E-01	6,94E-01
B total	PNR vs. Response	-0,15	0,08	6,35E-02	1,14E-01
B total	Grey zone vs. Response	**-0,22**	0,08	8,26E-03	**2,48E-02**
B total	PNR vs. Remission	0,03	0,06	6,81E-01	7,00E-01
B total	Grey zone vs. Remission	0,00	0,07	9,83E-01	9,83E-01
B total	Response vs. Remission	**0,18**	0,07	1,37E-02	**3,28E-02**
F total	PNR vs. Grey zone	-0,14	0,08	9,93E-02	1,70E-01
F total	PNR vs. Response	0,08	0,09	3,56E-01	4,74E-01
F total	Grey zone vs. Response	0,20	0,09	2,88E-02	6,09E-02
F total	PNR vs. Remission	-0,04	0,07	5,27E-01	6,54E-01
F total	Grey zone vs. Remission	0,07	0,07	3,26E-01	4,51E-01
F total	Response vs. Remission	-0,17	0,08	3,26E-02	6,52E-02

Table S2. PANTS Consortium.
All UK gastroenterologists were invited to participate in the PANTS study, which was promoted through the UK National Institute for Health Service Research (NIHR) and the British Society of Gastroenterology (BSG). The PANTS Consortium consists of the following people (including those already listed as authors):

Hospital or Trust name	City	Name	Job Title
Tameside Hospital NHS Foundation Trust	Ashton U Lyne	Dr Vinod Patel	Consultant Gastroenterologist
Basildon and Thurrock University Hospitals NHS Foundation Trust	Basildon	Dr Zia Mazhar	Consultant Gastroenterologist
Hampshire Hospitals NHS Foundation Trust	Basingstoke	Dr Rebecca Saich	Consultant Gastroenterologist
Royal United Hospital	Bath	Dr Ben Colleypriest	Consultant Gastroenterologist
Ulster Hospital	Belfast	Dr Tony C Tham	Consultant Gastroenterologist
University Hospital's Birmingham NHS Foundation Trust	Birmingham	Dr Tariq H Iqbal	Consultant Gastroenterologist
East Lancashire NHS Teaching Trust	Blackburn	Dr Vishal Kaushik	Consultant Gastroenterologist
Blackpool Teaching Hospitals NHS Foundation Trust	Blackpool	Dr Senthil Murugesan	Consultant Gastroenterologist
Bolton NHS Trust	Bolton	Dr Salil Singh	Consultant Gastroenterologist
Royal Bournemouth Hospital	Bournemouth	Dr Sean Weaver	Consultant Gastroenterologist
Bradford Teaching Hospitals Foundation Trust - (St Lukes Hospital &Bradford Royal Infirmary)	Bradford	Dr Cathryn Preston	Consultant Gastroenterologist
Brighton and Sussex University Hospitals NHS Trust	Brighton	Dr Assad Butt	Paediatric Consultant Gastroenterologist
Brighton and Sussex University Hospitals NHS Trust	Brighton	Dr Melissa Smith	Consultant Gastroenterologist
University Hospitals Bristol NHS Foundation Trust	Bristol	Dr Dharamveer Basude	Consultant Paediatric Gastroenterologist
University Hospitals Bristol NHS Foundation Trust	Bristol	Dr Amanda Beale	Consultant Gastroenterologist
Frimley Park Hospital NHS Foundation Trust	Camberley	Dr Sarah Langlands	Consultant Gastroenterologist
Frimley Park Hospital NHS Foundation Trust	Camberley	Dr Natalie Direkze	Consultant gastroenterologist
Cambridge University Hospitals NHS Foundation Trust	Cambridge	Dr Miles Parkes	Consultant Gastroenterologist

Cambridge University Hospitals NHS Foundation Trust	Cambridge	Dr Franco Torrente	Consultant Paediatric Gastroenterologist
Cambridge University Hospitals NHS Foundation Trust	Cambridge	Dr Juan De La Revella Negro	Research fellow
North Cumbria University Hospitals NHS Trust	Carlisle	Dr Chris Ewen MacDonald	Consultant Gastroenterologist
Ashford & St Peter's Hospitals NHS Foundation Trust	Chertsey	Dr Stephen M Evans	Consultant Gastroenterologist
St Peter's Hospital	Chertsey	Dr Anton V J Gunasekera	Consultant Gastroenterologist
Ashford & St Peter's Hospitals NHS Foundation Trust	Chertsey	Dr Alka Thakur	Paediatric Consultant
Chesterfield Royal NHS Foundation Trust	Chesterfield	Dr David Elphick	Consultant Gastroenterologist
Colchester Hospital University NHS Foundation Trust	Colchester	Dr Achuth Shenoy	Consultant Gastroenterologist
University Hospitals Coventry and Warwickshire NHS Trust	Coventry	Prof Chuka U Nwokolo	Consultant Gastroenterologist
County Durham and Darlington NHS Foundation Trust	Darlington	Dr Anjan Dhar	Consultant Gastroenterologist & Hon. Clinical Lecturer
Derby Hospital NHS Foundation NHS Trust	Derby	Dr Andrew T Cole	Consultant Gastroenterologist
Doncaster and Bassetlaw Hospitals NHS Foundation Trust	Doncaster	Dr Anurag Agrawal	Consultant Gastroenterologist
Dorset County Hospital NHS Foundation Trust	Dorchester	Dr Stephen Bridger	Consultant Gastroenterologist
Dorset County Hospitals Foundation Trust	Dorchester	Dr Julie Doherty	Paediatric Consultant
Dudley Group NHS Foundation Trust	Dudley	Dr Sheldon C Cooper	Consultant Gastroenterologist
Russells Hall Hospital, The Dudley Group NHS Foundation Trust	Dudley	Dr Shanika de Silva	Consultant Gastroenterologist
Ninewells Hospital & Medical School	Dundee	Dr Craig Mowat	Consultant Gastroenterologist
East Sussex Healthcare Trust	Eastborne	Dr Phillip Mayhead	Consultant Gastroenterologist
NHS Lothian	Edinburgh	Dr Charlie Lees	Consultant Gastroenterologist and Honorary Senior Lecturer
NHS Lothian	Edinburgh	Dr Gareth Jones	Research fellow
Royal Devon and Exeter NHS Foundation Trust	Exeter	Dr Tariq Ahmad	Consultant Gastroenterologist
Royal Devon and Exeter NHS Foundation Trust	Exeter	Dr James W Hart	Consultant Paediatrician

Royal Devon and Exeter NHS Foundation Trust	Exeter	Dr Nicholas A Kennedy	Consultant Gastroenterologist
Royal Devon and Exeter NHS Foundation Trust	Exeter	Dr James R Goodhand	Consultant Gastroenterologist
Royal Devon and Exeter NHS Foundation Trust	Exeter	Dr Simeng Lin	Research fellow
Royal Devon and Exeter NHS Foundation Trust	Exeter	Dr Neil Chanchlani	Research fellow
Royal Devon and Exeter NHS Foundation Trust	Exeter	Rachel Nice	Research fellow
Royal Devon and Exeter NHS Foundation Trust	Exeter	Timothy J McDonald	Consultant clinical scientist
Royal Devon and Exeter NHS Foundation Trust	Exeter	Claire Bewshea	Group Manager
Glasgow Royal Infirmary	Glasgow	Dr Daniel R Gaya	Consultant Gastroenterologist
Royal Hospital for Children	Glasgow	Prof Richard K Russell	Consultant Paediatric Gastroenterologist
Royal Hospital for Children	Glasgow	Dr Lisa Gervais	Research fellow
Gloucestershire Hospitals NHS Trust	Gloucester	Dr Paul Dunckley	Consultant Gastroneterologist
United Lincolnshire Hospitals NHS Trust	Grantham	Dr Tariq Mahmood	Consultant Gastroenterologist
James Paget University Hospitals NHS Foundation Trust	Great Yarmouth	Dr Paul J R Banim	Consultant Gastroneterologist
Calderdale and Huddersfield NHS Trust	Halifax	Dr Sunil Sonwalkar	Consultant Gastroenterologist
Princess Alexandra Hospital NHS Trust	Harlow	Dr Deb Ghosh	Consultant Gastroenterologist
Princess Alexandra Hospital NHS Trust	Harlow	Dr Rosemary H Phillips	Consultant Gastroenterologist
Hull and East Yorkshire NHS Trust	Hull	Dr Amer Azaz	Paediatric Consultant Gastroenterologist
Hull and East Yorkshire NHS Trust	Hull	Dr Shaji Sebastian	Consultant Gastroenterologist
Airedale NHS Foundation Trust	Keighley	Dr Richard Shenderey	Consultant Gastroenterologist
Crosshouse Hospital	Kilmarnock	Dr Lawrence Armstrong	Consultant Paediatrician
Crosshouse Hospital	Kilmarnock	Dr Claire Bell	Research fellow
The Queen Elizabeth Hospital NHS Foundation Trust	Kings Lynn	Dr Radhakrishnan Hariraj	Consultant Gastroenterologist
Kingston Hospital NHS Trust	Kingston upon Thames	Dr Helen Matthews	Consultant Gastroenterologist
NHS Fife	Kirkcaldy	Dr Hasnain Jafferbhoy	Consultant Gastroenterologist
Leeds Teaching Hospitals NHS Trust	Leeds	Dr Christian P Selinger	Consultant Gastroenterologist
Leeds Teaching Hospitals NHS Trust	Leeds	Dr Veena Zamvar	Paediatric Consultant Gastroenteorlogist

University Hospitals of Leicester NHS Trust	Leicester	Prof John S De Caestecker	Consultant Gastroenterologist
University Hospitals of Leicester NHS Trust	Leicester	Dr Anne Willmott	Paediatric Consultant Gastroenterologist
Mid Cheshire Hospitals NHS Foundation Trust	Leighton	Mr Richard Miller	Research Nurse
United Lincolnshire Hospitals NHS Trust	Lincoln	Dr Palani Sathish Babu	Consultant Gastroenterologist
Alder Hey Childrens Hospital	Liverpool	Dr Christos Tzivinikos	Consultant Paediatric Gastroenterologist
University College London Hospitals NHS Foundation Trust	London	Dr Stuart L Bloom	Consultant Gastroenterologist
Kings College Hospital NHS Foundation Trust	London	Dr Guy Chung-Faye	Consultant Gastroenterologist
Royal London Childrens Hospital, Barts Health NHS Trust	London	Prof Nicholas M Croft	Paediatric Consultant Gastroenterologist
Chelsea & Westminster Hospital	London	Dr John ME Fell	Consultant Paediatric Gastroenterologist
Chelsea and Westminster Hospital NHS Foundation	London	Dr Marcus Harbord	Consultant Gastroenterologist
North West London Hospitals NHS Trust	London	Dr Ailsa Hart	Consultant Gastroenterologist
Kings College Hospital NHS Foundation Trust	London	Dr Ben Hope	Consultant Paediatrician
Guys & St Thomas' NHS Foundation Trust	London	Dr Peter M Irving	Consultant Gastroenterologist
Barts and The London NHS Trust	London	Prof James O Lindsay	Consultant Gastroenterologist
Guy's and St Thomas' NHS trust	London	Dr Joel E Mawdsley	Gastroenterology Consultant
Lewisham and Greenwich Healthcare NHS Trust	London	Dr Alistair McNair	Consultant Gastroenterologist
Chelsea and Westminster Hospital NHS Foundation	London	Dr Kevin J Monahan	Consultant Gastroenterologist
Royal Free London NHS Foundation Trust	London	Dr Charles D Murray	Consultant Gastroenterologist
Imperial College Healthcare NHS Trust	London	Prof Timothy Orchard	Consultant Gastroenterologist
St George's Healthcare NHS Trust	London	Dr Thankam Paul	Paediatric Consultant Gastroenterologist
St George's Healthcare NHS Trust	London	Dr Richard Pollok	Reader and Consultant Gastroenterologist
Great Ormond Street Hospital for Children NHS Foundation Trust	London	Dr Neil Shah	Consultant Gastroenterologist
North West London Hospitals NHS Trust	London	Dr Sonia Bouri	Research fellow
The Luton & Dunstable University Hospital	Luton	Dr Matt W Johnson	Consultant Gastroenterologist

Luton and Dunstable Hospital Foundation Trust	Luton	Dr Anita Modi	Paediatric Consultant with Allergy and Gastroenterology interest
The Luton & Dunstable University Hospital	Luton	Dr Kasamu Dawa Kabiru	Research fellow
Maidstone and Tunbridge Wells NHS Trust	Maidstone	Dr B K Baburajan	Consultant Gastroenterologist
Maidstone and Tunbridge Wells NHS Trust	Maidstone	Prof Bim Bhaduri	Paediatric Consultant Gastroenterologist
Manchester University Hospitals NHS Foundation Trust	Manchester	Dr Andrew Adebayo Fagbemi	Consultant Gastroenterologist
Central Manchester University Hospitals NHS Foundation Trust	Manchester	Dr Scott Levison	Consultant Gastroenterologist
The Pennine Acute Hospitals NHS Trust	Manchester	Dr Jimmy K Limdi	Consultant Gastroenterologist
Manchester University NHS Foundation Trust, Wythenshawe Hospital	Manchester	Dr Gill Watts	Consultant Gastroenterologist
Sherwood Forest Hospitals NHS Foundation Trust	Mansfield	Dr Stephen Foley	Consultant Gastroenterologist
South Tees Hospital NHS Foundation Trust	Middlesbroug h	Dr Arvind Ramadas	Consultant Gastroenterologist
Milton Keynes Hospital NHS Foundation Trust	Milton Keynes	Dr George MacFaul	Consultant Gastroenterologist
Newcastle Upon Tyne Hospital Trust	Newcastle	Dr John Mansfield	Consultant Gastroenterologist
Isle of Wight NHS Foundation Trust	Newport	Dr Leonie Grellier	Consultant Gastroenterologist
Norfolk & Norwich University Hospital NHS Foundation Trust	Norwich	Dr Mary-Anne Morris	Consultant Paediatric Gastroenterologist
Norfolk & Norwich University Hospital NHS Foundation Trust	Norwich	Dr Mark Tremelling	Consultant Gastroenterologist
Nottingham University Hospitals NHS Trust	Nottingham	Prof Chris Hawkey	Consultant Gastroenterologist
Nottingham University Hospitals NHS Trust	Nottingham	Dr Sian Kirkham	Consultant Paediatric Gastroenterologist
Nottingham University Hospitals NHS Trust	Nottingham	Dr Charles PJ Charlton	Consultant gastroenterologist
Oxford University Hospitals NHS Foundation Trust	Oxford	Dr Astor Rodrigues	Paediatric Consultant Gastroenterologist
Oxford University Hospitals NHS Trust	Oxford	Prof Alison Simmons	Consultant Gastroenterologist
Plymouth Hospitals NHS Trust	Plymouth	Dr Stephen J Lewis	Consultant Gastroenterologist
Poole Hospital NHS Foundation Trust	Poole	Dr Jonathon Snook	Consultant Gastroenterologist
Poole Hospital NHS Foundation Trust	Poole	Dr Mark Tighe	Paediatric Consultant with interest in Oncology and Gastroenterology

Portsmouth Hospitals NHS Trust	Portsmouth	Dr Patrick M Goggin	Consultant Gastroenterologist
Royal Berkshire NHS Foundation Trust	Reading	Dr Aminda N De Silva	Consultant Gastroenterologist
Salford Royal NHS Foundation Trust	Salford	Prof Simon Lal	Consultant Gastroenterologist
Shrewsbury and Telford Hospital NHS Trust	Shrewsbury	Dr Mark S Smith	Consultant Gastroenterologist
South Tyneside NHS Foundation Trust	South Shields	Dr Simon Panter	Consultant Gastroenterologist
Southampton University Hospitals NHS Trust	Southampton	Dr JR Fraser Cummings	Consultant Gastroenterologist
Southampton University Hospitals NHS Trust	Southampton	Dr Suranga Dharmisari	Research fellow
East and North Herts NHS Trust	Stevenage	Dr Martyn Carter	Consultant Gastroenterologist
NHS Forth Valley	Stirling	Dr David Watts	Consultant Gastroenterologist
Stockport NHS foundation Trust	Stockport	Dr Zahid Mahmood	Consultant Gastroenterologist
North Tees and Hartlepool NHS Foundation Trust	Stockton	Dr Bruce McLain	Paediatric Consultant Gastroenterologist
University Hospitals of North Staffordshire	Stoke-on Trent	Dr Sandip Sen	Consultant Gastroenterologist
University Hospitals of North Midlands NHS Trust	Stoke-on-Trent	Dr Anna J Pigott	Consultant Paediatric Gastroenterologist
City Hospitals Sunderland NHS Foundation Trust	Sunderland	Dr David Hobday	Consultant Gastroenterologist
Taunton and Somerset NHS Foundation Trust	Taunton	Dr Emma Wesley	Consultant Gastroenterologist
South Devon Healthcare NHS Foundation Trust	Torquay	Dr Richard Johnston	Consultant Gastroenterologist
South Devon Healthcare NHS Foundation Trust	Torquay	Dr Cathryn Edwards	Consultant gastroenterologist
Royal Cornwall Hospitals NHS Trust	Truro	Dr John Beckly	Consultant Gastroenterologist
Mid Yorkshire Hospitals NHS Trust	Wakefield	Dr Deven Vani	Consultant Physician & Gastroenterologist
Warrington& Halton NHS Foundation	Warrington	Dr Subramaniam Ramakrishnan	Consultant Gastroenterologist
West Hertfordshire Hospitals NHS Trust	Watford	Dr Rakesh Chaudhary	Consultant Gastroenterologist
Sandwell and West Birmingham Hospitals NHS Trust	West Bromwich	Dr Nigel J Trudgill	Consultant Gastroenterologist
Sandwell and West Birmingham Hospitals NHS Trust	West Bromwich	Dr Rachel Cooney	Consultant gastroenterologist
Weston Area Health NHS Trust	Weston-Super-Mare	Dr Andy Bell	Consultant Gastroenterologist
Royal Albert Edward Infirmary, Wrightington,	Wigan	Dr Neeraj Prasad	Consultant Gastroenterologist

Wigan & Leigh NHS Foundation Trust			
Hampshire Hospitals NHS Foundation Trust	Winchester	Dr John N Gordon	Consultant Gastroenterologist
Royal Wolverhampton Hospitals NHS Trust	Wolverhampto n	Prof Matthew J Brookes	Consultant Gastroenterologist
Western Sussex Hospitals NHS Trust	Worthing	Dr Andy Li	Consultant Gastroenterologist
Yeovil District Hospital NHS Foundation Trust	Yeovil	Dr Stephen Gore	Consultant Gastroenterologist
